# Head and neck cancer patient journey’s health literacy: a multidisciplinary team perspective. VOICE study

**DOI:** 10.1007/s00520-023-08256-7

**Published:** 2024-01-27

**Authors:** Ana Joaquim, Cláudia Vieira, Leonor Ribeiro, Anabela Barros, Inês Leão, Cecília Alvim, Sara Pinheiro, Mafalda Nogueira, Catarina Morais

**Affiliations:** 1 Centro Hospitalar Vila Nova de Gaia E Espinho, Vila Nova de Gaia, Portugal; 2 IPO Porto, Porto, Portugal; 3 Centro Hospitalar Universitário Lisboa Norte, Lisboa, Portugal; 4 Centro Hospitalar Universitário de Coimbra, Coimbra, Portugal; 5 MSD Portugal, R. da Qt. da Fonte 19, 2770-192 Paço de Arcos, Portugal

**Keywords:** R/M HNSCC, Multidisciplinary teams, Patients’ and caregivers’ information needs

## Abstract

**Purpose:**

Health literacy is a current Public Health priority in Portugal. The participation of well-informed patients in their care and shared decision making are essential, especially in chronic aggressive and debilitating pathologies such as recurrent or metastatic (R/M) Head and Neck Squamous Cell Carcinoma (HNSCC).

**Aims:**

This study aimed to characterize R/M HNSCC patients’ and caregivers’ information needs identified by healthcare professionals (HCPs).

**Methods:**

Two online Focus Groups, one with only medical doctors and the other with other HCPs involved in the treatment of R/M HNSCC patients, were conducted, using a modified Metaplan, Lean or adapted PDCA methodology. The discussions were audio recorded in full and content analysis was performed using ATLAS.ti qualitative data analysis software.

**Results:**

Topics addressed were diagnosis, treatment, quality of life, and global evaluation. In general, all experts agreed that only essential information should be cautiously given, according to patients’ and caregivers’ wishes. It was consensual that patients are given the necessary information to adhere to treatment. Two main barriers were identified: one barrier was associated with verbal communication due to the lack of health literacy of these patients, and the other barrier regarded healthcare access. It was also considered important to remind patients of the daily and social activities that they could and should maintain, as well as providing sufficient social resources and problem-solving training to caregivers.

**Conclusions:**

This qualitative study highlights the complexity of R/M HNSCC patients’ care. Immediate availability of psychologists and psychiatrists should be implemented in all centers that treat HNSCC patients. The differences found between the physicians' Focus Group and other HCPs’ Focus Group in some of the addressed topics emphasize the importance of a multidisciplinary and holistic approach, in a biomedical model integrated with a biopsychosocial model.

**Supplementary Information:**

The online version contains supplementary material available at 10.1007/s00520-023-08256-7.

## Introduction

Health literacy is a current Public Health priority in Portugal. The low health literacy level of the Portuguese population has individual and social costs that hamper patients’ autonomy and decrease the Quality of Life of both patients and caregivers [1]. The participation of well-informed patients in their care and shared decision making is essential [1], especially in chronic aggressive and debilitating pathologies such as recurrent or metastatic (R/M) Head and Neck Squamous Cell Carcinoma (HNSCC). This is because these patients face multiple comorbidities, disease and treatment related complications, that are managed through complex and multidisciplinary care settings [2].

The Portuguese General Directorate of Health states that patients with higher health literacy have higher adherence to therapy, better self-management of disease, and a higher ability to participate in decisions regarding their condition. In order to achieve this, relevant and credible information has to be provided [1].

Although receiving a diagnosis of cancer is inevitably distressing, appropriate communication between healthcare providers and patients is known to alleviate some elements of the traumatic nature of this experience. Providing comprehensive and relevant information can assist the patients by preparing them and enhancing their ability to manage their illness. Moreover, empirical data indicate that patients’ perceptions of adequate information and support are predictive of positive rehabilitation outcomes in the 2- to 6-year post-treatment period [3]. On the other hand, inadequate information seems to increase stress and leads to mistrust [4].

It has been reported that the impact of treatment on social activities and interactions of patients with R/M HNSCC is under-discussed and a key concern. Also, patients appear to be given information regarding survivorship topics, such as psychological well-being and patient support groups, less frequently than information concerning disease and treatment [5]. Additionally, there is a significant gap in addressing communication and informational needs of caregivers and family members who are integral for promoting healthy behaviors and self-care post-treatment [3].

Providing tailored information to R/M HNSCC patients using different styles seems feasible and helps in simplifying the complex information without compromising the quality and quantity [4]. Healthcare providers should recognize the information needs patients and provide them with the required information in a format that aids understanding [4].

This study aimed to characterize R/M HNSCC patients’ and caregivers’ information needs identified by healthcare professionals (HCPs), including timing and formats for health literacy initiatives. 

## Methods

This study was based on two online Focus Groups, both occurring in May 2022. A structured sample was recruited to ensure the participation of different HCPs from different types of hospitals.

Both focus groups followed an adapted methodology based on modified Metaplan [6, 7], Lean [8–11] or adapted PDCA [12–14] methodology, as follows: the facilitator asked each question to each participant, who shared their opinion with all the other members, without previously writing it on a post-it for subsequent discussion. At the end of each group of questions, the facilitator opened the discussion.

The discussion of each focus group was audio recorded in full and the contents of these recordings were transcribed. Each data report from each focus group was subject to pre-specified thematic analysis of content by an expert researcher. This analysis was performed using the ATLAS.ti qualitative data analysis software [15]. Answers were grouped in common themes and expressed as numbers and percentages. Given the small sample size per focus group, no sub-group analysis was performed.

In order to perform content analysis of the Focus Groups using ATLAS.ti, different codes were applied, with the aim to summarize the opinion of the participants in the Focus Groups. These codes were integrated into four themes based on previously defined questions concerning patient journey: diagnosis, treatment, Quality of Life, and Global Evaluation – Table S1.

The analysis encompassed two steps. In the first step, data were analyzed on a semantic level, using an inductive, bottom-up analysis focused on the prevalence of the data, in which the relevant information was assigned to codes based on topics. Therefore, the coders were able to summarize the data and be more familiar with the text and relevant patterns of information, to simplify the next step of the analysis – categorical codes (Table S2). In the second step, a top-down, deductive analysis was performed, and these codes were integrated into the four themes reported in Table S1.

In order to quantify the most important topics, some codes were altered or eliminated, and some were sub-divided into more specific ones. Through this analysis, all important topics were included and analyzed in terms of frequency.

The final text was agreed upon on August 8, 2023. All authors contributed and were actively involved in the preparation of this manuscript.

## Results

In total, 12 participants with 1 to 30 years of experience in R/M HNSCC multidisciplinary teams, were included in the study. In Focus Group 1, only medical doctors were included: 3 medical oncologists, 1 radio-oncologist, 1 general surgeon, and 1 otolaryngologist. In Focus Group 2, other HCPs involved in the treatment of R/M HNSCC patients were included: 2 nurses, 1 physical therapist, 1 psychologist, 1 speech therapist, and 1 nutritionist. Each group had HCPs from 3 types of Hospitals – Cancer Institute, University Hospital and General Hospital.

Four main themes were addressed: diagnosis, treatment, quality of life, and global evaluation (Table S1). The codes assigned to participants’ discourse, followed by the number and percentage of participants that addressed each topic, are described in Table 1 (Diagnosis), Table 2 (Treatment), Table 3 (Quality of Life), and Table 4 (Global evaluation).

**Table 1:** Diagnosis: topics addressed by the participants

Focus Group 1: medical specialists			Focus Group 2: other healthcare professionals		
Information (n / %)					
According to the patient´s prior knowledge	6	100	Caregivers are not prepared; burn-out of the caregiver	6	100
Tumor staging and disease extension	6	100	Functional alterations	6	100
According to the patients/caregivers wishes	6	100	Excessive information given to the patient	6	100
Information about other necessary medical appointments	3	50	Only necessary and essential information	6	100
Results of previous exams	3	50	According to the patient/caregiver wishes	6	100
All the information	2	33.3	Patient journey	6	100
Simplified language	1	16.7	According to the patient prior knowledge	5	83.3
			Type of treatment	2	33.3
			Clarification of patients' doubts	1	16.7
			Disease severity, prognosis, and survival	1	16.7
			How to convey the disease to family members	1	16.7
Who gives the information? (n / %)					
The physician who first suspected of the disease	6	100	The physician who first suspected of the disease	6	100
Medical Oncologists and/or Radiotherapists	3	50			
Supporting materials (n / %)					
Nurses’ contact	6	100	Visual support: anatomical images	6	100
Assistant doctor’s contact	6	100	Excessive information given to the patient	6	100
Flyer: Head and Neck	6	100	Videos	6	100
Videos	2	33	Easy access to healthcare professionals	6	100
Material for patients with communication difficulties	1	16.7	Flyers: useful as complementary	5	83.3
			Flyers: social and financial support	2	33.3
			Provide contact of other patients and patient associations	2	33.3
Difficulties and barriers (n / %)					
Access to Health Care including delay in diagnosis, delay in exam scheduling, delay in patient referral, delay in the availability of the results of exams	6	100	Emotional barrier	6	100
Patient’s education level	6	100	Verbal Communication	6	100
Communication with the patient	4	66.7	Lack of health literacy	6	100
			Immediate follow-up: psychology and psychiatry	5	83.3
			Social barriers	4	66.7
			Feeding and swallowing	4	66.7
			Organization of the consultations	3	50.0
			Social support	2	33.3
			Continuous rehabilitation	2	33.3
			Financial shortfall	2	33.3
			Refusal of psychological support	1	16.7
			Family barriers	1	16.7

**Table 2:** Treatment: topics addressed by the participants

Focus Group 1: medical specialists			Focus Group 2: other healthcare professionals		
Information (n / %)					
Support consultations	6	100	Functional alterations	6	100
Adverse events	6	100	Feeding and swallowing	6	100
Only necessary and essential	6	100	According to the patient and caregiver wishes	6	100
Goal and benefit	6	100	Lack of health literacy	6	100
Duration and frequency	5	83.3	Only necessary and essential information	6	100
Patient’s circuit	4	66.7	According to the patient´s prior knowledge	5	83.3
Multidisciplinary component	4	66.7	Adverse events	5	83.3
			Treatment impact	5	83.3
			Chronicity of treatment effects	3	50.0
			Multidisciplinary component	2	33.3
			Surgery type	1	16.7
			Manage expectations	1	16.7
Who gives the information? (n / %)					
Treating physician	6	100	The physician who first suspected of the disease	6	100
Supporting materials (n / %)					
Nurses’ contact	6	100	Visual support: anatomical images	6	100
Assistant doctor’s contact	6	100	Videos	6	100
Treatment flyer	6	100	Flyers: useful as complementary	6	100
Administrative’s contact	3	50	Nutritional supplements	3	50.0
			Personalized plan	2	33.3
			Contact with different materials used to overcome functional alterations	1	16.7
Adherence (n/ %)					
Patients have enough information regarding treatment	6	100	Patients have enough information regarding treatment	6	100
Burn-out of caregiver and home support	6	100	Excessive information given to the patient	6	100
Patient’s education level	6	100	Lack of health literacy	6	100
Alcoholic habits	4	67	Caregivers are not prepared; burn-out of the caregiver	6	100
Healthcare optimization	3	50	Only necessary and essential information	6	100
			According to the patient´s prior knowledge	5	83.3
Resources of the institution regarding treatment (n / %)					
The institution provides all the necessary treatments	6	100	The institution provides all the necessary treatments	3	50.0
Support consultations	6	100	Lack of human resources	3	50.0
Lack of human resources	6	100	Access to Health Care including delay in diagnosis, delay in exam scheduling, delay in patient referral, delay in the availability of the results of exams	1	16.7
Multidisciplinary component	5	83			
Palliative care	3	50			
Nurse	2	33.3

**Table 3:** Quality of life: topics addressed by the participants

Focus Group 1: medical specialists			Focus Group 2: other healthcare professionals		
Information (n / %)					
Support consultations	6	100	Functional alterations	6	100
Caregivers are not prepared; burn-out of the caregiver	6	100	Limitations in verbal communication due to the disease	6	100
Adverse events	6	100	Feeding and swallowing	6	100
Only necessary and essential information	6	100	How to maintain a social life	2	33.3
According to the patient´s wishes	6	100			
Provide contact of other patients and patient associations	3	50.0			
How to maintain a social life	3	50.0			
Resilience	2	33.3			
Dependency level and the need for a family readjustment	1	16.7			
Supporting materials (n / %)					
			Personalized plan	3	50.0

**Table 4:** Global evaluation: topics addressed by the participants

Focus Group 1: medical specialists			Focus Group 2: other healthcare professionals		
The support is getting better	4	66.7	Lack of time	6	100
It is ideal, but not possible	3	50	It is ideal, but not possible	5	83.3
Necessary home support	3	50	Lack of human resources	4	66.7
Necessary social support	3	50	Yes. we meet the goals for these patients	1	16.7
Necessary clinical trials	2	33.3			
Lack of time	2	33.3			
Better supporting material	2	33			
Yes, we meet the goals for these patients	1	16.7			

### Diagnosis

Table 1 shows the main codes assigned to participants’ discourse regarding information, supporting materials and barriers at diagnosis of R/M HNSCC. Medical professionals considered that, at diagnosis of R/M HNSCC, patients must be informed about tumor stage and extension of disease. Nevertheless, all participants agreed that this should be done integrating the patient´s prior knowledge and according to the patients’/caregivers’ wishes:

"(...) what is the patient’s will, according to our assessment of the patient, not in a very direct or literal way, but understanding what information the patient wants to receive, because there are patients who want to receive a lot of information about the diagnosis, the recurrence, the metastasis, and others who are a little more reluctant, this is also something that we have to evaluate and evaluate during the consultation (...)”

The other healthcare professionals also stressed the importance of caregivers in this process:

“(…) the information given is always what the patient and the family members, therefore the caregivers, want to know (…)”

Medical professionals highlighted the need of flyers, specifically for head and neck cancer:

“(…) informative booklet that is given to all patients in the first consultation by our nurses, who talk a little about the organization of the outpatient clinic, provide contacts of the emergency physician, the direct number they have to call, provide the direct number to our nurses, who organize and manage the appointments, and also talk a little about the side effects that are expected and that are common to most cancer treatments, they talk about the warning signs that patients should be aware of, talk a little about general care, and provide some information on food issues that is always a great doubt for patients. Unfortunately, this booklet/leaflet is not specific to head and neck patients, that is, it is a general booklet that is given to all cancer patients.”

Other HCPs considered that flyers can be useful as complementary information. However, visual support with anatomical images to explain the alterations that can occur are very important:

“(…) use of supporting images, anatomical images, explaining what anatomical and functional changes are to be expected. Then, I can adjust and adapt the explanation according to the difficulties the patient is having and the needs of the patient. (...)”

The delay in diagnosis and in access to exams are considered the main barriers for medical doctors:

“(…) the delay in the scheduling of exams or sometimes even in the report of the exams themselves. Sometimes they are even carried out within the ideal timing, but we do not always have the report available in time to expedite and define the subsequent strategy (…) Sometimes we may also have this difficulty in the delay in referral, that is, sometimes the diagnosis is a later diagnosis because most of our patients are admitted via the Emergency Service (…).”

On the other hand, for other HCPs, emotional and verbal communication barriers are often important limitations in this type of patients:

“(…) I think the emotional barriers of the patients themselves, sometimes our own barriers, because we cannot convey, or we cannot simplify the information so that the information is clear to the patients (…)”

### Treatment

Table 2 shows the main codes assigned to the participants’ discourse regarding the information and supporting materials to use during the treatment of R/M HNSCC. Medical professionals considered that it was important to talk to patients about the goal of treatment, toxicities, and support consultations:

“(…) the purpose of the treatment, that is, palliative or curative, in this case it is always palliative, duration of treatment, frequency of treatment, main toxicities, eventually available supportive therapy, contacts of the institution and all other support consultations, whether psycho-oncology, psychiatry, nutrition, or social worker, to inform the patient of all this multidisciplinary component existing in the institution, so that patients understand that all their different needs are covered (…).”

For the other HCPs, functional changes and impact of the treatment on feeding and swallowing ability should be explained to the patient and caregiver:

“(…) implications in functionality, for example, tissue fibrosis, edema, muscle rigidity, xerostomia, changes of dental arch, loss of teeth, all these have huge consequences in feeding and swallowing (…)”

For medical doctors, the most important support during treatment are the contacts details of nurses, the attending physician, and administrative services. However, other HCPs also added that, besides the supporting materials already identified in the diagnosis phase, the availability of nutritional supplements / samples of thickeners or ready-made foods (free of charge) would be of the utmost importance for these patients:

“(…) most of the patients require supplements, therefore, food, dietary supplements free of charge are important. For patients who cannot be fed orally, that is, who have nasogastric tubes or PEG, formulas, and supplements available in the market are not subsidized by the state. Many of these patients have economic difficulties (…)”

All participants agreed that patients with R/M HNSCC have sufficient information to be able to adhere to the proposed treatment. However, their education level and lack of literacy could negatively impact treatment adherence:

“(…) these are patients with a low level of literacy, and some of the caregivers also; patients frequently have a history of marked alcoholism, and there is a deficit, I mean, I do not say that there is a cognitive deficit, but we sometimes have patients who have some difficulty in understanding the message that is conveyed to them (…).”

The barriers identified by the participants are the same reported in Table 1 for the diagnosis.

### Quality of life

Table 3 includes the main points regarding the information that should be given to patients with R/M HNSCC on the impact of the disease and treatment on their quality of life. Besides functional alterations, participants highlighted the importance of caregivers at this point. Caregivers are not prepared for their role and risk burn-out:

“(…) caregivers are not prepared, it is also a challenge for them and often it is even a greater challenge because they have never had training for this and will not only be caregivers, they also have to manage the suffering resulting from the family member's disease (…)”

"(…) And we must also inform the caregivers that there is the caregiver's exhaustion and that they also have the right to have support and the right to be tired (...).”

Regarding quality of life, it could be important to provide the contact details of other patients and patient associations. They could give clues to these patients on how to maintain a social life with this disease:

“(…) show a little bit of what is the daily life of a patient with these conditions and with a recurrent or metastatic disease (...). ”

### Global evaluation

This section aims to provide an overview of the current support that healthcare professionals feel they are able to offer to R/M HNSCC patients in daily clinical practice (Table 4). Overall, the participants considered that support given to R/M HNSCC is getting better; however, it is still not ideal, mainly due to lack of time and human resources:

“(…) every day we fight to improve the delivery of our care, whether in diagnosis, treatment, articulation between different teams. I think there is always room for improvement, no doubt (...)”

“(...) I would sometimes like to have more time for each appointment, but with almost more than twenty patients on a morning…, I would like to have more time to talk and better manage the things that happen to these patients (...)”

“(...) we have a deficit of human resources (...)”.

**Fig. 1:**
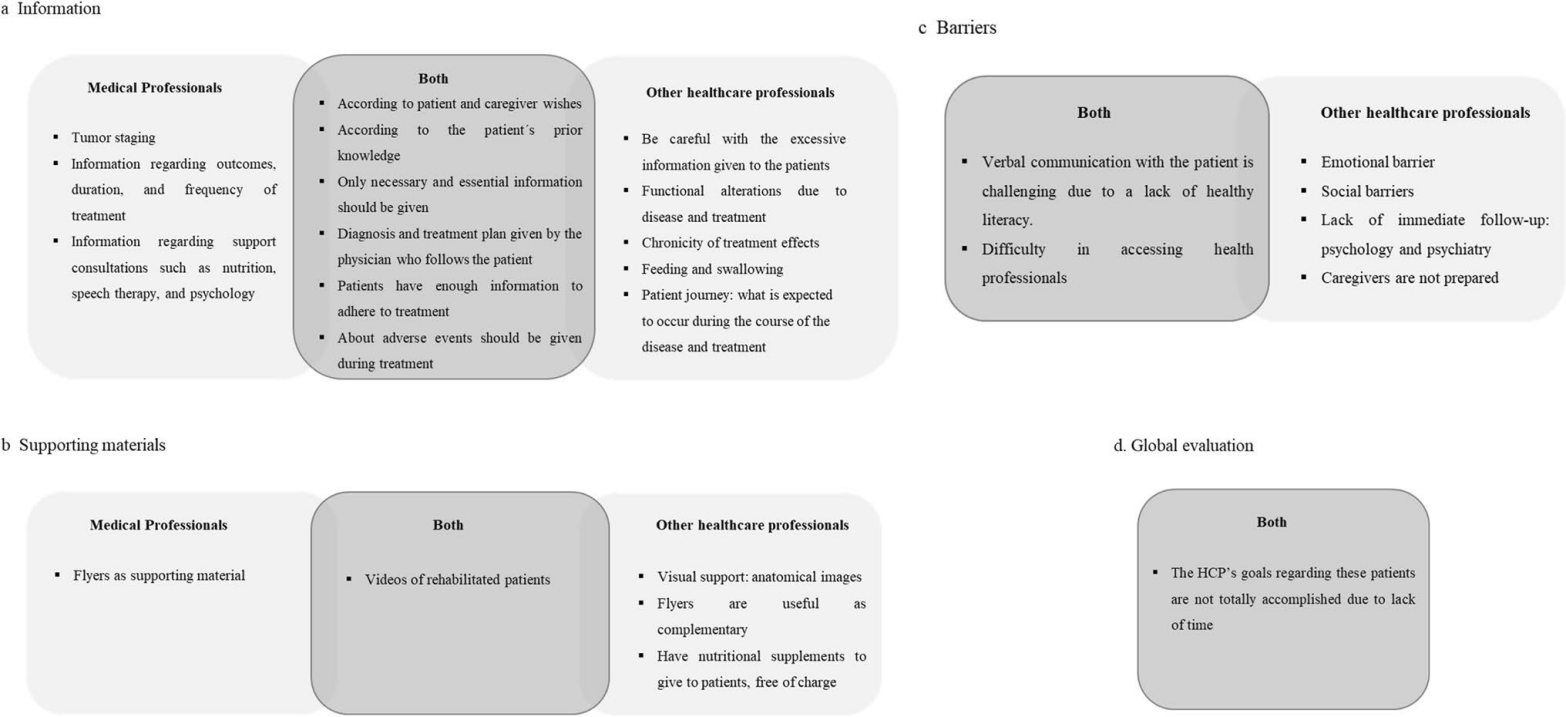
Comparison between the topics addressed in Focus Group 1 (Medical Professionals) and Focus Group 2 (other healthcare professionals). **a** Information; **b** Supporting materials; **c** Barriers: Participants from Focus Group 1 did not identify any barriers that were only mentioned by this group, resulting in a missed category for medical professionals; **d** Global Evaluation: there were no differences between the topics addressed by the two healthcare professionals groups; therefore, only a box with their collective opinion is included

A comparison between the topics addressed by the medical professionals and the other HCPs showed that some topics were only addressed by one of the Focus Groups. This comparison is shown in Fig. 1.

## Discussion

All healthcare professionals who participated in this study were experienced in the management of patients with R/M HNSCC, both in outpatient consultations and active participation in multidisciplinary team meetings. Therefore, their experience and opinion regarding R/M HNSCC patient journey are fundamental to understanding what is being offered and what can be improved in the follow-up of these patients.

All experts agreed that the main information regarding the diagnosis and treatment of R/M HNSCC patients should be given by the physician who first suspected the disease and follows-up those patients, respectively. This information should be given at diagnosis and at treatment decision, respectively. However, HCPs also highlighted that only essential information should be cautiously given, according to the patients’ prior knowledge and to patients’ and caregivers’ wishes.

It was consensual that patients are given the necessary information to adhere to treatment. However, it is not the only factor that impacts treatment adherence rates; although patients have all the needed information, other barriers, such as social and financial barriers, might contribute to patients’ missing treatments.

Two main barriers were identified: one associated with verbal communication due to the lack of health literacy of these patients, and the other one regarding healthcare access. To help in the management of these patients, videos of rehabilitated patients would be helpful. Finally, most HCPs agreed that they were not able to accomplish all the goals set for the follow-up of these patients.

To better understand the main concerns of the HCPs groups regarding the follow-up of these patients, an in-depth discussion of the identified topics was conducted. Physicians approached disease related topics, namely tumor stage, and specific treatment information. Moreover, they considered that disease and treatment specific flyers could be important as support materials for these patients. Other HCPs considered that flyers could be important but only as complementary information and should not include excessive information. In addition, these HCPs proposed other types of materials should be considered as supportive care, such as free of charge nutritional supplements. These last two points could be important to overcome the emotional and social barriers that these professionals consider to be important in the care of R/M HNSCC patients.

Emotional disturbance has been described in patients with head and neck cancers, not only at diagnosis, but also during the long treatment period [16]. Different actions should be taken to overcome this issue, namely inculcating a positive attitude and faith in the physician/treatment, expressing their emotions to family and friends, and indulging in activities to divert attention [16]. The latter was briefly addressed in both Focus Groups, i.e., it is important to remind patients of the daily and social activities which they could and should maintain. Friends and family of these patients may struggle to comprehend both the patients and their condition. Furthermore, the physical impact of the disease and its treatments can result in patients isolating themselves, making it challenging to find suitable activities. In light of this, healthcare professionals emphasize the importance of reminding the patients about the daily and social activities they can engage in to help overcome these challenges.

Another qualitative study with specialists in head and neck cancer also reported psycho-oncological support access as a barrier in head and neck cancer care [17]. There are behavioral risk factors associated with head and neck cancer that, together with treatment effects, could lead to psychosocial distress, social isolation, and lack of social support [18]. Regarding this topic, participants in Focus Group 2 also considered that an immediate follow-up with a psychologist or a psychiatrist should be available for these patients, and a social worker should also be considered. However, participants recognize that this may be distant from the current reality, and it is acknowledged that there is work to be done to align with these needs.

The role and burn-out of caregivers were also addressed. Although it was considered by HCPs to be a very important topic, few reports are found in the literature. Caregivers could experience poor psychological health and anxiety symptoms, mainly associated with patient cancer recurrence, ability to provide ongoing care, quality of life, and burn-out [19]. Therefore, it is of the utmost importance to provide sufficient social resources and problem-solving training to caregivers, helping them to positively cope with the situation and enhance family function [20].

Analysis of Focus Group 1 (physicians) showed that the physicians’ main goal was to treat the patient by targeting the disease, using a biomedical model that focuses mainly on biological factors. On the other hand, Focus Group 2 had a more patient-centered approach, with proximity to the patient, aiming to take care of the patient as a whole, including the social and psychosocial components, in an integrated biopsychosocial model [21]. The differences found in this qualitative study reinforce the importance of a multidisciplinary and holistic approach to treat and care for R/M head and neck cancer patients.

The main limitation of this study concerns the lack of patients’ and caregivers’ Focus Groups. Unfortunately, these Focus Groups were not conducted due to the COVID-19 pandemic. Concerns related to the virus led to reluctance among patients and caregivers to participate in in-person meetings. Additionally, the authors determined that conducting virtual sessions was not feasible due to the limited technological literacy within this population. Thus, we were not able to analyze their opinions and, together with HCPs opinions, draw conclusions regarding an R/M HNSCC patient journey in Portugal. Also, since the two Focus Groups were not face-to-face meetings, participants could be biased by the previous answers of their peers. However, the opinion of the HCPs could provide insights and help to develop ideas or hypotheses for future quantitative research.

## Conclusion

This qualitative study highlights the complexity of the R/M HNSCC patients’ care. Due to the behavioral risk factors associated with the disease, such as alcoholic habits and the low levels of R/M HNSCC patients’ health literacy, information should be given with caution throughout all patient journey steps. Furthermore, immediate availability of psychologists and psychiatrists should be implemented in all centers that treat HNSCC patients, to help overcome some social and emotional barriers, leading patients to adopt different behaviors and, consequently, a better adherence to treatment. The differences found between the physicians' Focus Group and other HCPs’ Focus Group in some of the addressed topics emphasize the importance of a multidisciplinary approach, in a biomedical model integrated with a biopsychosocial model.

### Supplementary Information

Below is the link to the electronic supplementary material.

Supplementary file1 (DOCX 57 KB)

## Data Availability

Not applicable.
