# Supplementary material for: Head and neck cancer patient journey’s health literacy: a multidisciplinary team perspective. VOICE study
Source: Support Care Cancer. 2024 Jan 27;32(2):133. doi: 10.1007/s00520-023-08256-7 (PMC10821956; doi:10.1007/s00520-023-08256-7)
Supplement: Supplementary file 1 — Supplementary file1 (DOCX 57 KB) [file 520_2023_8256_MOESM1_ESM.docx]

**TITLE:** Head and Neck Cancer Patient Journey’s Health Literacy: a multidisciplinary team perspective. VOICE study.

**APPENDIX**

**Table S1 -** Full Codebook: themes

| Themes | Definition |
| --- | --- |
| Diagnosis | Information to be given during the process of diagnosis: which, when, by whom, and how. Supporting materials. Difficulties and barriers. |
| Treatment | Information to be given during treatment: which, when, by whom, and how. Supporting materials. Difficulties and barriers. Patients’ needs and treatment adherence. |
| Quality of Life | Information to be given concerning the impact on the patients’ quality of life: which, when, and by whom. Supporting materials. |
| Global evaluation | HCPs’ expectations about patients’ care |

**Table S2** – Full Codebook: Categorical codes.

| **Codes** | **Definition** |
| --- | --- |
| Access to Healthcare including delay in diagnosis, delay in exam scheduling, delay in patient referral, delay in the availability of the results of exams | A barrier in the management of R/M HNSCC includes proper access to healthcare |
| According to the patient’s prior knowledge | The information that each HCP gives to each patient should be given after the HCP understands what is already known by the patient |
| According to the patient´s/ caregiver´s wishes | The information that each HCP gives to each patient/caregiver should be given after the HCP understands what the level of detail of the information is that the patient/caregiver wants to know |
| Administrative´s contact | The patients need to have direct contact with the administrative staff that can schedule medical/nurse appointments |
| Adverse events | Adverse events associated with the treatment |
| All the information | The information should not be adapted to each patient; all the information regarding diagnosis and treatment should be given to the patient |
| Caregivers are not prepared; burn-out of the caregiver | Caregivers are not prepared to support and accompany a patient with R/M HNSCC and eventually burn-out due to all the procedures that have to be performed on the patients 24/7 |
| Chronicity of treatment effects | Explain to the patient that some treatment effects are chronic |
| Clarification of patients' doubts | It is important to allow for the patient to ask questions and explain their doubts |
| Communication with the patient | It is difficult to convey the information to the patient |
| Supporting Materials: Contact with different materials used to overcome functional alterations | It would be important to have different medical materials, such as tracheostomy cannula and nasogastric tube |
| Continuous rehabilitation | These patients are in continuous rehabilitation and therefore there is an overload for these HCPs |
| Dependency level and the need for a family readjustment | It is important to explain to the patient what their level of dependency will be and the need for a family readjustment |
| Disease severity, prognosis, and survival | Disease severity, prognosis, and survival should be clearly explained to the patient |
| Duration and frequency | The duration and the frequency of the treatment should be carefully explained to the patient |
| Easy access to healthcare professionals | The patient needs to have easy access to HCPs |
| Emotional barrier | It is difficult to gain the confidence of the patient and it is difficult for the patient to accept the diagnosis |
| Excessive information to the patient | It is important to be careful regarding the amount of information given to the patient |
| Family barriers | The family does not support the patient, sometimes due to alcoholic habits |
| Feeding and swallowing | Barriers associated with the ability of feeding and swallowing |
| Financial shortfall | The financial conditions of these patients could limit their ability to buy some supplements that are not reimbursed |
| Flyers: social and financial support | These patients have many doubts about social and financial support. It could be included in a flyer |
| Flyers: useful as complementary | Flyers should not contain all information; they should be complementary to the information conveyed in other ways |
| Functional alterations | It is important to explain which functional alterations could occur due to the presence of the tumor, surgery, or treatment |
| Give contact of other patients and associations of patients | The contact with other patients and associations of patients will help the patient to feel better, supported and accompanied |
| Goal and benefit | The goal and benefit of the treatment should be carefully explained to the patient |
| How to maintain a social life | It is fundamental to explain to the patient how a social life could be maintained regardless of the limitations |
| How to convey the diagnosis to family members | Patients have concerns about how they should convey the diagnosis to their family |
| Immediate follow-up: psychology and psychiatry | There is a need to have immediate support from a psychologist or a psychiatrist |
| Information about other necessary medical appointments | Information about other medical appointments that the patient will need |
| It is ideal, but not possible | It is ideal to meet all the needs of these patients, however, it is not possible |
| Lack of health literacy | Lack of health literacy (patients and caregivers) is a barrier |
| Lack of human resources | There is a lack of human resources that could impact the optimization of the process during the treatment of the patient |
| Lack of time | There is a lack of time by HCPs |
| Limitations in verbal communication due to the disease | The localization of the tumor together with the treatment´s effect could impair the verbal capacity |
| Manage expectations | It is very important to manage the patient´s expectations, namely regarding treatment and rehabilitation |
| Medical Oncologists and/or radiotherapists | The information should be given by medical oncologists and/or radiotherapists |
| Multidisciplinary component | Explain to the patient that different HCPs will follow them during their journey |
| Necessary clinical trials | There is a need to increase the number of clinical trials including these patients |
| Necessary home support | There is a need to improve home support |
| Necessary social support | There is a need to improve social support |
| Nurse | Each patient should have a nurse that accompanies the patient during the entire process |
| Nutritional supplements | It would be important to give the patients nutritional supplements free of charge |
| Only necessary and essential | The amount of information given to a patient should be carefully selected: only necessary and essential information should be given |
| Organization of the consultations | Consultations in the hospitals are not properly organized, it is difficult to manage the available time for these patients |
| Palliative care | Institutions have palliative care available |
| Patient circuit | The circuit that the patient has in the hospital should be carefully explained |
| Patient journey | Explain the expected patient journey |
| Patient’s education level | Most patients with R/M HNSCC have a low education level and addictive behaviors that could contribute to the difficulties in communication with the patient and adherence to treatment |
| Refusal of psychological support | Patients refuse psychological support |
| Resilience | It is important to talk with the patient about the need to be resilient |
| Results of previous exams | Information about the results of the previous medical exams |
| Simplified language | A simplified and adapted language should be used when HCPs give information to a patient or a caregiver |
| Social barriers | Barriers associated with the way of living |
| Social support | There is a lack of human resources that could give social support to these patients |
| Support consultations | R/M HNSCC patients need support consultations: Nutrition, Psychology, among others |
| Supporting Material: Flyer – Head and Neck | A specific flyer of Head and Neck instead of a general flyer about oncologic diseases |
| Supporting Material: Material for patients with communication difficulties | Material helping patients with communication limitations due to the disease or the treatment should be provided aiming to facilitate the integration of the patient |
| Supporting Material: Videos | Videos showing rehabilitated patients can be shown to the patient |
| Supporting materials: Assistant doctor´s contact | The patients need to have direct contact with their assistant doctor |
| Supporting materials: Nurse contact | The patients need to have direct contact with a nurse |
| Supporting Materials: Personalized plan | A personalized plan should be established for each patient |
| Supporting Materials: Samples of thickeners or ready-made foods | It is important to have samples of different thickeners or ready-made foods in the hospital, for the patients to try in the presence of HCPs |
| Supporting Materials: Treatment Flyer | A flyer with complementary information regarding treatment would be important |
| Surgery type | Explain the surgery that will be performed |
| The institution provides all the necessary treatments | All the necessary treatments are provided by each institution |
| The physician who first suspected of the disease | The information given to patients suspected to have R/M HNSCC should be conveyed by the physician who first suspected of the disease, not necessarily an oncologist, and asked for medical exams to confirm the diagnosis |
| The support is getting better | The support given to these patients is getting better |
| Treating physician | Physicians that will treat the patient should inform the patient regarding the specificities of the treatment |
| Treatment adherence: Alcoholic Habits | The maintenance of alcoholic habits during the treatment could lead the patient not to adhere to treatment |
| Treatment adherence: Burn-out of caregiver and home support | The burn-out of the caregiver could impact treatment adherence if the caregiver is unable to accompany the patient’s hospital visits |
| Treatment adherence: Healthcare optimization | The complexity of the patient´s circuit in a specific visit to the hospital in which the treatment is going to be administrated could limit treatment adherence |
| Treatment adherence: Patients have enough information regarding treatment | There is no lack of information concerning treatment that can impact the adherence of the patient to the treatment |
| Tumor staging and disease extension | Information about the stage of the tumor and the extension of the disease |
| Type of treatment | Explain the type of treatment |
| Verbal Communication | Verbal communication due to a lack of health literacy |
| Visual support: anatomical images | Showing anatomic images will help the patient to understand what is happening and what is going to happen with the treatment |
| Better supporting material | To accomplish all the goals, better supporting materials are needed |
| Yes, we meet the goals for these patients | We are meeting all the needs of these patients |
